# Cervical spine injury in the young child

**DOI:** 10.1007/s00586-012-2292-1

**Published:** 2012-06-26

**Authors:** Navin N. Ramrattan, F. Cumhur Öner, Bronek M. Boszczyk, Rene M. Castelein, Paul F. Heini

**Affiliations:** 1 Centre for Spinal Studies and Surgery, Queen’s Medical Centre, Nottingham University Hospitals NHS Trust, Nottingham University Hospitals, West Block D Floor, Derby Road, Nottingham, NG7 2UH UK; 2 Department of Orthopaedic Surgery, University Medical Centre Utrecht, PO Box 85500, 3508 GA Utrecht, The Netherlands; 3 Department of Spinal Surgery, Klinik Sonnenhof, Buchserstrasse 30, 3006 Bern, Switzerland

**Keywords:** Cervical spine injury, Cervical spine clearance, Spinal cord injury, Pediatric, Trauma

## Abstract

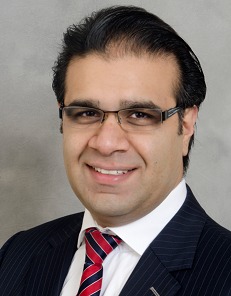

This Grand Rounds is about the clinical and radiological presentation, treatment and outcome of pediatric cervical spine injury. A 15-month-old girl suffers from a motor vehicle accident and is intubated on-site because of progressive agitation. Whole body trauma CT was read as normal. When sedation was discontinued after 24 h she was found to be tetraplegic below C6 level. MRI shows a total disruption between C6 and C7 that in hindsight was also visible on the initial trauma CT. She was treated surgically by an anterior and posterior reconstruction and was post-operatively treated with a halo vest. Clearing the cervical spine in young children is deceptively difficult. Meticulous review and interpretation of conventional radiographs and CT are important yet MRI should be considered in uncertain cases. Severe ligamentous injury without concomitant bony injury occurs more frequently than in older children and adults, with sometimes devastating consequences.

## Case presentation

A 15-month-old baby girl was thrown out of the baby seat when the car her parents were driving collided with another vehicle at moderate speed in wintery conditions. One parent suffered from rib fractures and the other was unharmed. The baby girl was unresponsive to pain, was whimpering and becoming progressively agitated and had a Glasgow Coma Score of 6. Because of suspicion of brain trauma, the baby was transferred to an academic centre for further diagnostic work-up after intubation. There on admission, she underwent regular trauma screening with no obvious injury detected to the spine and was admitted to the intensive care unit. After 24 h of sedation, however, she was found to be tetraplegic below neurological level C6.

## Diagnostic imaging

**Fig. 1:**
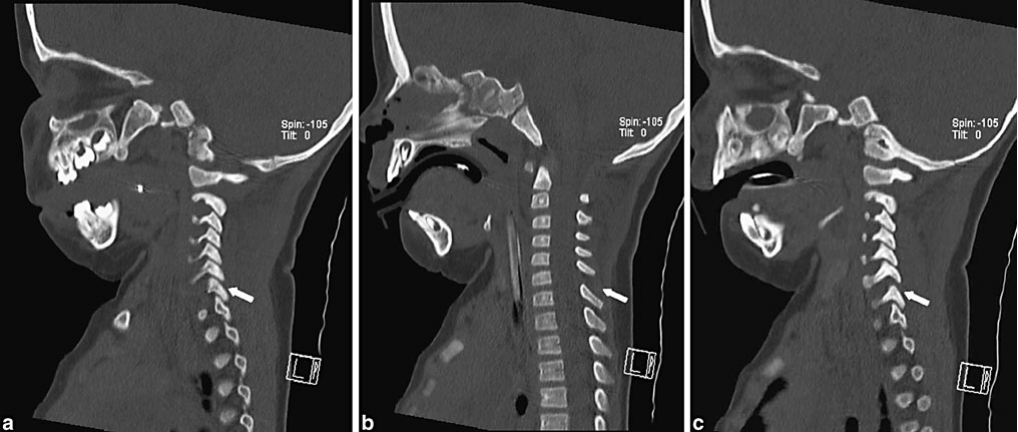
Sagittal reconstruction of the initial CT of the cervical spine. A normal alignment and distance is seen at facet joints between C6 and C7 *on the left* (**a)** as opposed to a diastasis and subluxed state of the facet joint at the same level *on the right* (**c**) The midline sagittal reconstruction (**b)** shows an increased distance between the interspinous processes of C6 and C7 indicating an injury to the interspinous ligament at that level. The alignment between C6 and C7 is still normal

**Fig. 2:**
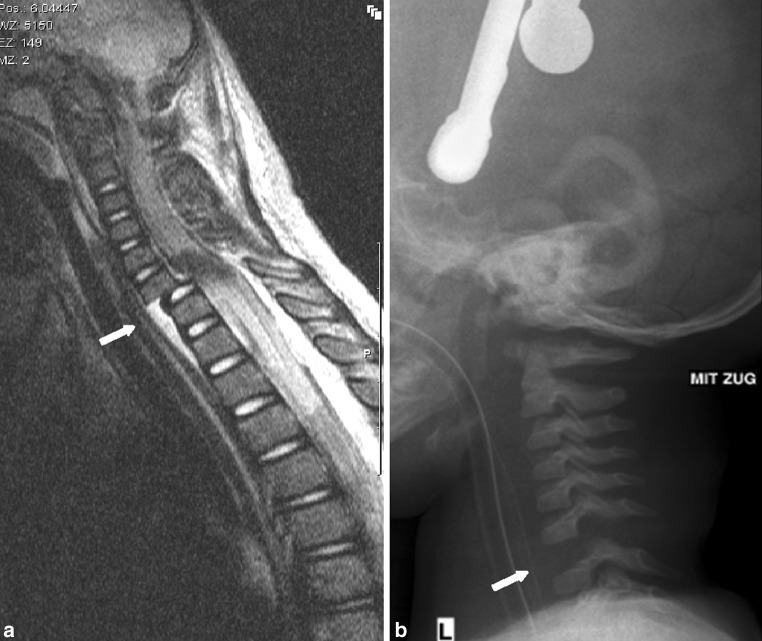
**a** T2 weighted MRI images after the spinal cord injury was detected clinically, showing a separation of the vertebral endplate from the vertebral body through the growth plate of C6 (typical for children) and the subsequent hematoma lifting the anterior longitudinal ligament off the vertebral body of C7 and Th1. There is a translation of C6 in relation to C7. Inhomogeneity of the signal in the spinal cord indicates severe spinal cord injury. Inhomogeneity of the signal at the interspinous ligament between C6 and C7 indicates an injury. **b** Radiograph under slight traction already showing a clear diastasis between vertebral body of C6 and C7 indicating complete disruption of the discoligamentous complex

At the time of presentation in the emergency department, a CT scan of the brain—including the cervical spine—and thorax and abdomen were performed and read as normal by the radiologist. At that time, no MRI was performed. In hindsight, the distraction injury to the cervical spine is visible on the CT scan (Fig. 1). When, after discontinuation of sedation, the tetraplegia was diagnosed, MRI revealed the spinal injury (Fig. 2a). A radiograph under slight traction, with the intention of spinal realignment, further illustrates the complete disruption of the discoligamentous complex.

## Review of the literature

Historically, the literature specifically addressing cervical spine injuries in children has been scarce; most studies have been focused on adults. In recent years, as distinct aspects of the pediatric spine have been appreciated, more attention has been given exclusively to injuries of the cervical spine in younger patients. Challenges still remain in the completion of knowledge regarding pediatric cervical injuries, their management and the development of suitable surgical hardware for very young patients [1].

### Epidemiology

Injuries of the cervical spine are relatively rare in children but there is significant potential for considerable morbidity when it does occur. In the United States, the incidence of all spinal cord injuries in the pediatric population is 18.1 spinal cord injuries per 1 million children per year, representing approximately 1,300 new cases each year [2]. Sixty to eighty percent of spine injuries in children occur at the cervical level, in contrast to adults where 30–40 percent occur at the cervical level. The most common mechanisms of injury include not only motor vehicle accidents and falls but also auto-pedestrian accidents, bicycle mishaps, obstetrical complications, sports, child abuse and diving accidents account for many injuries. Falls are more common in younger children, whereas motor vehicle accidents are more common in older children [1, 3]. Only 1 in 60,000 births are associated with spinal cord injuries, with the upper cervical region most frequently involved.

Cervical spine injuries in young children are a distinct clinical entity with a different distribution of injuries as compared with those found in adults [1]. Children younger than 8 years of age usually have upper cervical injuries, while children older than 8 years more frequently have lower cervical injuries, a pattern more adults alike [4]. This is in accordance with the biomechanical properties of the developing cervical spine as opposed to the spine nearing maturity [2].

Younger children have increased flexibility of the cervical spine. This increase in mobility is explained by incomplete ossification of the vertebral bodies, ligament laxity and incomplete development of the spinous processes. In addition, the immature spine has a more horizontal orientation of the facet joints. When combined with an increase in head-to-torso ration and relatively weak cervical musculature, this increase in flexibility leads to cervical spine injuries at higher levels.

### Diagnosis

The malleability of the pediatric cervical spine not only leads to a different distribution of injuries but also to distinct radiographic features. In addition, in children, it is possible to injure the spinal cord and that display objective signs of myelopathy without suffering an obvious injury to the bony spine: termed spinal cord injury without radiographic abnormality (SCIWORA) on CT and plain films [1, 5].

Interpretation of radiographs of the cervical spine of young patients with cervical trauma can be challenging [6]. Pseudosubluxation is commonly seen in children under 8 years of age. The amount of subluxation should be less than 4.0 mm and should be corrected with extension. The atlanto-dens space can be larger in children (up to 4.0 mm), and variations in odontoid development can commonly be misinterpreted as an injury on plain cervical radiographs. Soft tissue spaces can also appear enlarged in a crying and uncooperative child [7].

In contrast to adults and adolescents, CT imaging of the cervical spine in children younger than 8 years of age likely does not have as much utility and often exposes the child to unnecessary radiation [2]. New generation multidetector scanners expose the child to increased doses of radiation, and there is an increased risk of iatrogenic cancer with increased exposure. In particular, exposure of the thyroid during cervical spine scanning is worrisome. CT scanning of the cervical spine is very sensitive for osseous injuries and fracture. Unfortunately, most children in this age group sustain more ligamentous injuries than osseous injury. Therefore, the lower sensitivity of CT scanning does not justify the increased risk of radiation exposure in this population, especially not for screening purposes [8].

In a review of over 600 children under the age of 5 years, 147 had CT imaging to clear the cervical spine in addition to plain radiographs, and 4 osseous injuries were identified. In all four, the injury was found on the plain radiograph and no new injuries were identified by CT [9].

MRI is highly sensitive and the clinical relevance of many of the subtle findings on MRI has not been completely established. Often, obtaining a cervical spine MRI requires deep sedation or general anesthesia takes much longer and may require transport of potentially unstable patients to areas far from the emergency department or intensive care unit. MRI, however, identifies spinal cord injury as well as soft tissue and ligamentous injury particularly well, both of which are poorly identified on a CT scan. In infants and children, MRI may be the study of choice for clearing the difficult cervical spine [2].

Evaluation of a protocol established to obtain an MRI on all children that could not be cleared clinically or with plain radiographs or CT within 72 h revealed a trend toward reduced intensive care unit and hospital days, as well as a savings of 7.700 dollars per patient [10].

**Fig. 3:**
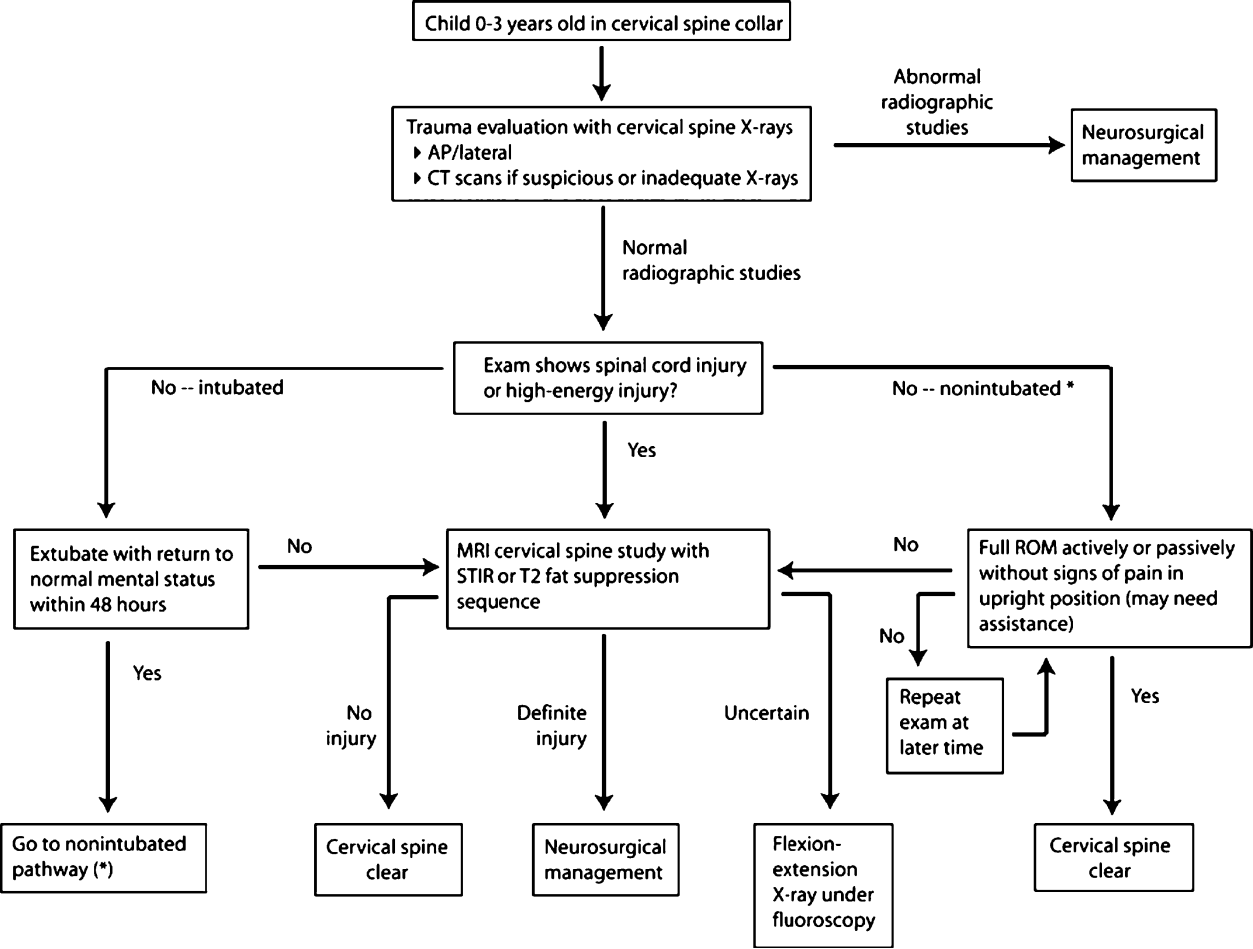
Flow diagram of cervical spine clearance in non-communicative children, *AP* anteroposterior; *X-ray* radiograph. Reproduced with permission [12]

Young children are not able to communicate effectively and severe injuries may require intubation and sedation. Cervical spine clearance after trauma in children between 0 and 3 years is therefore deceptively difficult. Recently, however, three proposed algorithms for cervical spine clearance in children have been published [2, 11] including one specifically for non-communicative infants (Fig. 3) [12]. These guidelines attempt to integrate NEXUS (=National Emergency X-Radiography Utilization Study) criteria, cervical spine radiographs, neurological status and additional imaging studies like CT, MRI and flexion/extension radiographs. Even though NEXUS criteria are valid only for adults and inapplicable in non-communicative infants or children younger than 3-year old, they may aid in clinical decision making concerning elder children [13].

In a retrospective analysis of pooled data from two level one pediatric trauma centers utilizing the spine clearance protocol shown in Fig. 3, 575 cervical spines of children under 3-year old were cleared in a 5-year period. Authors report that in addition to plain radiographs (100 %) in 14 % CT studies and in 10 % MR scans were obtained in order to clear the cervical spine. Very rarely (no exact number is mentioned) flexion–extension studies under fluoroscopy were necessary. Twenty-eight injuries (4.9 %) were detected: 19 ligamentous, 4 fractures with or without other injuries and 5 dislocations with or without other injuries. Four patients required operative stabilization. No late (initially missed) injuries were detected. Unfortunately, no data are provided specifying how many injuries were found on CT that had not been identified on radiographs already. No data were provided on the distribution of patients in to groups in which MR only (e.g. in case of adequate but clearly abnormal radiographs rather than suspicious radiographs no CT is obtained), CT only or both MR and CT were obtained. It was quite likely though, that most patients for whom an MR was to be obtained had already had a CT scan. Also, many of the patients about to undergo a CT scan would also require a MR later . Kreykes et al. [2] advocate a spine clearance algorithm for young children in which CT scanning is abandoned altogether, based on the supposition that CT scanning has a very low likelihood finding injuries not found on plain film and reducing radiation exposure in this population is a clinical priority.

In our case, the initial CT scan already shows an increased distance between the spinous processes of C6–C7 and in one of the facet joints between C6 and C7. Given the absence of reliable physical examination in the intubated child and the suspicious findings, an MRI would be indicated within 48 h according to the protocol of Anderson et al. [12] or within 72 h according to the algorithm as proposed by Kreykes et al. [2]. The MRI that was obtained after 24 h showed translation of C6 in relation to C7 with an obvious spinal cord lesion in accordance with clinical findings of a tetraplegic child from neurological C6 level and below. There is a clear injury of discoligamentous complex (DLC) with subsequent hematoma anterior of the vertebrae C7, Th1 and Th2. (Note the synchondrosis in C2 between dens and corpus on the initial CT).

### Pathology

In the current case, the history is indicative of a high energy trauma in which it is unclear which forces exactly had affected the cervical spine. On the MRI, the intervertebral disc is ruptured from the vertebral body indicating a severe anterior ligament injury as a result of hyperextension. In addition, the posterior ligamentous complex must be severed as well as the capsule of the facet joints in order to permit the observed translation between C6 and C7. Spinal instability is certain with likely continuous cord compression in a setting of proven neurologic deficit. This results in a SLIC Score (subaxial injury of the cervical spine) well over the threshold in which surgical stabilization is indicated [14].

### Differential diagnosis

A multitude of differential diagnoses can be mentioned, among the following. A subaxial fracture, SCIWORA, head trauma with neurological deficit and a lesion of the vertebral artery, pre-existent myelopathy, a pre-existent neurological disease especially of the spinal cord and of peripheral nerves, congenital anomaly, persisting apophysis and synchondroses, pathological fracture.

### Rationale for treatment and evidence-based literature

The instability of the cervical spinal column in which the intervertebral disc is ruptured from the end-plate due to severe translation in the presence of a ruptured posterior ligament is too unstable to be treated with a halo vest only [14–16]. In addition, in this case in which there is tetraplegia in a child, the facilitation of nursing and the possibility of early mobilization have a high priority [17]. Based on the best available evidence, though of low quality, there is a strong recommendation for the use of instrumentation in unstable pediatric spinal fractures to allow for the realignment of the bony elements of the spinal column and protection of the neural elements [18].

As both the intervertebral disc and the posterior ligament complex are severely injured, a reconstruction from both anterior and posterior is indicated in order to reestablish both alignment and tension band [14].

Skeletal skull traction should be applied with caution in young children with cervical spine trauma, as it may be dangerous when an unstable lesion is accidentally overdistracted. Unstable injuries of the cervical spine can be substantially overdistracted with quite small weights [19]. It is difficult to predict which patient will develop traumatic vertical dissociation after being subjected to traction [20]. Hence traction, if at all indicated, should be performed with very moderate weight and ideally under fluoroscopic control.

## Procedure

**Fig. 4:**
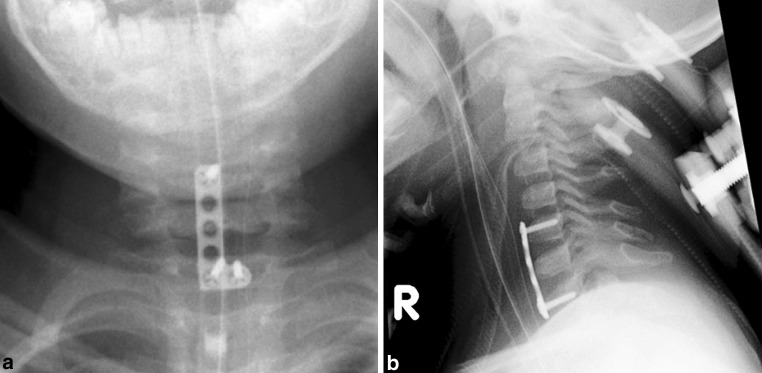
Procedure imaging section. **a**, **b** Postoperative radiographs showing the anterior reconstruction of the tension band using a modified T-plate and two screws distally in C7 and one screw proximally in C5. A posterior Ethibond cerclage was performed to augment the injured posterior ligament from C5 to C7

A pediatric halo ring was applied and the dislocation was reduced under fluoroscopy after which the halo ring was connected to the dorsal part of a halo vest providing some primary stability. A standard right-sided anteromedial approach to the cervical spine was performed. A prominent thymus gland was noted as well as residual diastasis at segments C6–C7. Also CSF flow was visible, indicating a dural tear. The anterior tension band was reconstructed by applying a small fragment modified T-plate from C5 to C7, after a 2 mm H plate turned out to be too short and insufficient purchase was achieved in C6. The T-plate was modified into an L-shaped plate by resecting one branch. Two screws were placed in C7, one screw in C5 and none in C6. The motion segments were bridged without fusion. The wound was closed, leaving a drain. The anterior part of the halo vest was applied and the posterior part was removed after the patient was repositioned into a prone position. The positioning was checked again under fluoroscopy. A midline dorsal approach was performed and a non-resorbable Ethibond cerclage was performed in the interspinous ligaments from C5 to C7. The spinous processes themselves were too cartilaginous to provide any support. The wound was closed and the halo vest was re-applied. No change in neurology was observed post-operatively (Fig. 4).

Three months post-operatively, the modified T-plate and screws were removed via an anterior approach. The stability of the cervical spine was found to be sufficient preoperatively.

## Outcome and follow-up

The patient was admitted to the paraplegic ward and transferred to the Netherlands where she has been followed-up for 6 years now in order to detect any spinal deformity early. She is now doing well, and after intensive rehabilitation even able to do some horseback riding. She is able to move the upper extremities and even able to write her own name.
